# The effect of oil palm‐dominated landscapes on the home range and distribution of a generalist species, the Asian water monitor

**DOI:** 10.1002/ece3.8531

**Published:** 2022-01-26

**Authors:** Sergio Guerrero‐Sanchez, Katherine Majewski, Pablo Orozco‐terWengel, Silvester Saimin, Benoit Goossens

**Affiliations:** ^1^ Organisms and Environment Division School of Biosciences Cardiff University Cardiff UK; ^2^ Danau Girang Field Centre, c/o Sabah Wildlife Department Kota Kinabalu Malaysia; ^3^ Sabah Wildlife Department Kota Kinabalu Malaysia; ^4^ Sustainable Places Research Institute Cardiff University Cardiff UK; ^5^ Present address: Institute of Borneo Studies University College Sabah Foundation Kota Kinabalu Malaysia

**Keywords:** anthropogenic landscape, Asian water monitor lizard, Borneo, GPS‐telemetry, spatial ecology, *Varanus salvator*

## Abstract

The Asian water monitor lizard, *Varanus salvator*, is one of the largest predators in Southeast Asia which persists in human‐dominated landscapes and, as such, is a suitable model to understand the behavioral plasticity of generalists in anthropogenic landscapes. We used Local Convex Hull with adaptive algorithm to estimate the home range size of 14 GPS‐tagged individuals, followed by a MAXENT approach and community prey composition to understand the habitat preferences within the landscape. We estimated larger home ranges in forest than in oil palm plantations, as well as a larger diversity and abundance of mammals. Core home ranges were always linked to water bodies. However, the use of underproductive oil palm, freshwater swamp forest, and degraded forest by monitor lizards were higher than other kind of vegetation. This suitable habitat is proportionally larger in forest (73.7%) than in oil palm plantations (39.6%). Generalized estimation equation models showed that, while full home range size was negatively associated with the abundance of mammals, core areas depicted a positive association with mammal abundance, as well as with the proportion of suitable habitat within the home range. Besides having smaller home ranges in oil palm plantations, our findings suggest that limited suitable habitat availability forces the Asian water monitor lizard's population to establish only one or very few core areas. Contrastingly, under the protection of forest, they have more core areas, widely dispersed within larger home ranges. We conclude that regardless the plasticity of the species, human‐dominated landscapes are altering natural patterns of home range establishment in the monitor lizard's population, creating a potential ecological trap where conditions may not remain favorable for them in the long run. A deeper understanding of the ecological implications on the species and the prey community is advisable.

## Graphical abstract

We use the Asian water monitor lizard as a model species to understand how oil palm‐dominated landscapes may alter natural patterns of home range in generalist species. We described how relevant is the role of the natural forest around (and within) oil palm plantations as source of protection and to facilitate the dispersion of the population within the landscape.

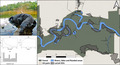

## 1 INTRODUCTION

Home range is defined as the area where an individual meets the necessary requirements to perform its ecological functions (Baker, 1978). The size and distribution of home range is determined by the existent environmental features on the landscape (Cristescu et al., 2016; Dyer et al., 2001; Houle et al., 2010). Human‐dominated landscapes usually increase localized food abundance, thereby promoting a reduction of the individuals' home range (Saïd & Servanty, 2005; Smith & Griffith, 2009). For example, Rajaratnam et al. (2007) suggest that the intensive use of oil palm plantations by leopard cats (*Prionailurus bengalensis borneensis*), also a common carnivore generalist in Borneo, is highly associated to high prey catchability and abundance. Harlow et al. (2010), however, highlight the fundamental role of other environmental variables for Komodo dragons' habitat selection (*V*. *komodoensis*), suggesting the preference of areas with dense vegetation, as they offer the most suitable thermal habitats with more stable temperature.

Home range reduction can translate into a sedentary behavior, with a more intensive use of resources in the area, including negative impacts on the dynamics of the prey community (Jessop et al., 2012; Smith & Griffith, 2009). Thus, understanding the home range and habitat preferences of generalist carnivores can provide information not only about the species plasticity in human‐dominated landscapes, but also regarding to the distribution and structure of prey communities, with implications for landscape management.

The Asian water monitor lizard (*Varanus salvator)* is one of the largest generalist carnivores in Southeast Asia, which persists in human‐dominated landscapes (Fitzsimons & Thomas, 2016; Traeholt, 1994; Uyeda, 2009). The extremely broad diet of the species is associated with a spatially large foraging area, where solitary individuals roam actively searching for live prey or carcasses for large portions of the day (Fitzsimons & Thomas, 2016; Karunarathna et al., 2017; Traeholt, 1994). However, in the Kinabatangan floodplain, a previous study suggests that the forest surrounding large extensions of oil palm plantations plays an important role on the stability of the population (Guerrero‐Sanchez et al., 2021), raising fundamental questions on the spatial ecology of the species.

Telemetry has provided useful information for species distribution, enabling researchers to estimate the habitat size needed by certain species to satisfy their requirements, such as protection, nutrition, reproduction, and gene flow (i.e. Hearn et al., 2018; Sastrawan & Ciofi, 2002; Stark et al., 2017). It has also been helpful to understand how resources are used and distributed within the landscape, in order to predict when and where certain species may and may not occur (Bastille‐Rousseau et al., 2016; McCue et al., 2014). Very high frequency (VHF) telemetry has been used to study various species of varanids (i.e. Auffenberg, 1981; Bennett, 2014; Ciofi et al., 2007), with few of them focusing on *V*. *salvator* (Gaulke et al., 1999; Traeholt, 1995, 1997). In contrast, GPS technology has only been used in two studies on *V*. *varius* (Flesch et al., 2009; Lei & Booth, 2018), despite its substantial advantages regarding to the accuracy and the volume of data generated (Hebblewhite & Haydon, 2010; Kochanny et al., 2009; Tomkiewicz et al., 2010).

This study aims to understand the spatial dynamics of a scavenger species, the Asian water monitor lizard, in the complex human‐modified landscape of the Kinabatangan floodplain. Specifically, we aimed to (1) estimate home range sizes in both forested areas and oil palm plantations, (2) identify the environmental variables determining the distribution of the monitor lizards' population within the study area, and (3) assess the composition of prey communities existing within the home ranges. We predicted that Asian monitor lizards have smaller home ranges when inhabiting oil palm habitats because of the higher abundance of prey and the limited distribution of habitat that provides suitable refugia.

## 2 METHODS

### 2.1 Study site

**FIGURE 1:**
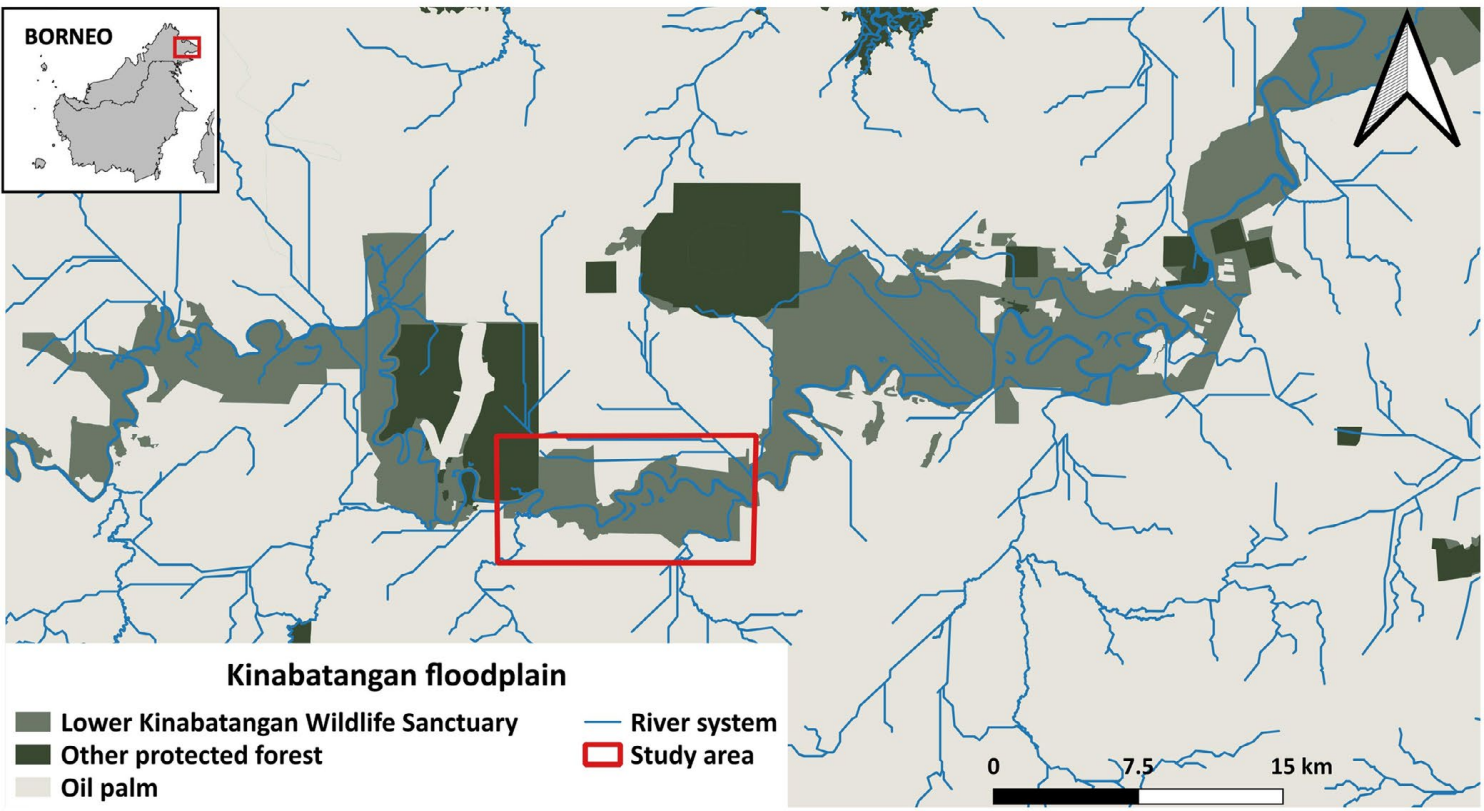
Delimited study area within the Kinabatangan floodplain

The study was carried out within the Kinabatangan floodplain, in Sabah, East Malaysia. The landscape consists of a complex matrix of varying forest types mixed with rural settlements and large extensions of oil palm (*Elaeis guineensis*) plantations along the Kinabatangan River. Oxbow lakes, tributaries, and streams irrigate the landscape either seasonally or permanently (Abram et al., 2014; Estes et al., 2012) (Figure 1). The area offers the opportunity to understand how species persist within a severely degraded landscape (i.e. Goossens et al., 2016; Hearn et al., 2018; Stark et al., 2017).

### 2.2 Telemetry data collection

Twenty adult monitor lizards (*n*_Oil palm_ = 10; *n*_Forest_ = 10), heavier than 15 kg, were tagged with VHF/GPS backpack‐like devices (Advanced Telemetry Systems Inc., North Isanti, MN USA) between January 2015 and December 2016. In order to minimize the effect of territoriality, lizards tagged within the same period of time were trapped with a minimum distance of 2 km from each other. All the lizards tagged were trapped using wired‐mesh cage traps (*L* = 90 cm, *W* = 40 cm, *H* = 40 cm), and baited with chicken entrails (Guerrero‐Sanchez et al., 2021).

GPS‐Tags were slightly modified from the VHF‐tags described by Ciofi et al. (2007) and Harlow et al. (2010) for Komodo dragons. The backpack‐like devices consisted of a block of waterproof resin that wrapped four different elements: (1) a GPS sensor to record the lizard daily movements, (2) a VHF transmitter to identify the current location of the individual on the ground, (3) an ultra‐high frequency UHF transmitter that allows the device to communicate with the base‐station and transmits the collected information from the device (including battery status), and (4) two “AA” alkaline batteries.

**FIGURE 2:**
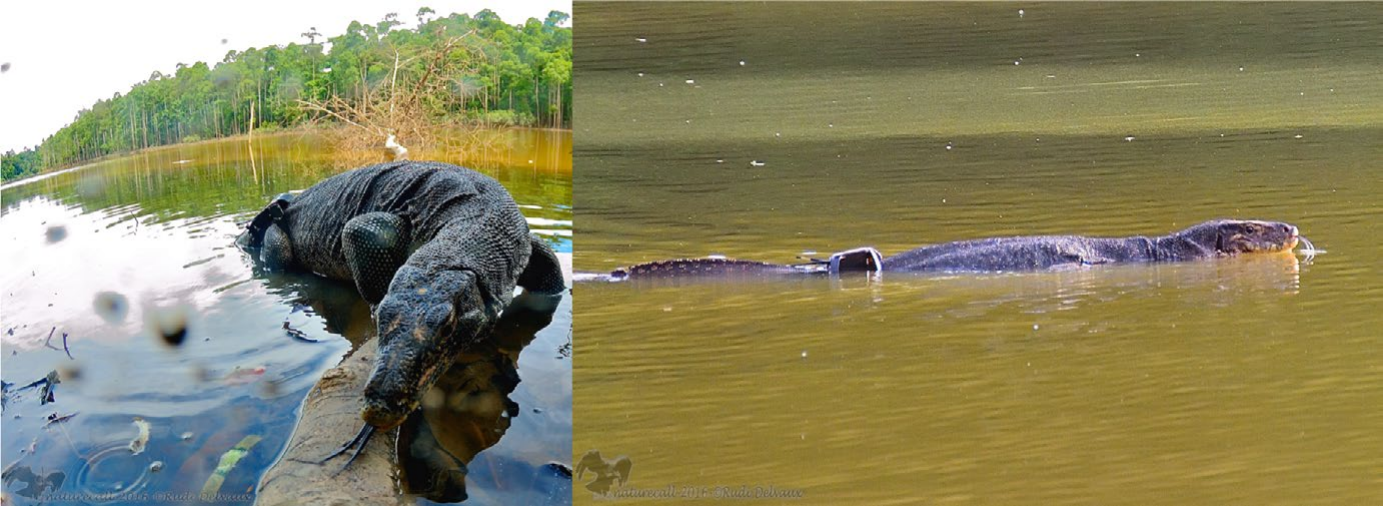
Asian water monitor lizard in an oxbow lake, in the Lower Kinabatangan Wildlife Sanctuary. A VHF/GPS backpack‐like device is attached onto the hip

Although the weight of a tag was only ~65 g, its dimensions and attaching system permitted us to deploy it only on individuals above 15 kg, as it may have slipped off from smaller individuals (Figure 2). A more detailed description of the GPS trackers performance can be found in the Appendix S1.

The tracking schedule was fixed to record one GPS location every 90 min. from 05:00 until 20:00 h. every day, while the VHF/UHF system was set to operate daily from 07:00 until 12:00 h. These settings would allow the tags to work from 4 to 9 months, depending on environmental conditions and canopy density. Independence among consecutive GPS locations was assumed with the 90‐min. interval (Ciofi et al., 2007). Data were downloaded once a week, but lizards were VHF‐tracked every other day for the first 2 weeks to confirm that tags had been properly attached and that the animals did not show any injury associated with the attachment. In order to prevent any health hazards related to long‐term tag attachment, all the tags were retrieved from the lizards before the batteries fully lost their charge.

### 2.3 Home range estimation

To maximize accuracy in home range estimation, we only included individuals whose home ranges showed no variation during at least two consecutive weeks. Home ranges were calculated on AdehabitatHR v. 0.4.15 for R (Calenge, 2006), using the local convex hull estimation with adaptive algorithm (a‐LoCoH). One of the advantages of LoCoH estimations is that the algorithm allows to shape an accurate home range by considering physiographic features such as rivers, lakes, and cliffs (Getz et al., 2007; Huck et al., 2008; Kie et al., 2010). The estimated core area was represented by 50% of the GPS locations, while a buffer area was defined by 75% of the recorded GPS locations, and the transient zone (full home range) included up to 95% of the observations (Ciofi et al., 2007; Huck et al., 2008; Kie et al., 2010).

### 2.4 Home range predictors

Although monitor lizards have frequently been recorded around both still and running water sources (Traeholt, 1995; Uyeda, 2009), relatively little is known about how the Asian water monitor lizard perceives aquatic and terrestrial features on the landscape. Therefore, the exact landscape characteristics, or combination of characteristics, which monitor lizards identify as refugia, are unknown. In order to address this, a wide breadth of environmental data available for the study site was gathered to identify the spatial niche of the population. We used two categorical and three continuous variables to represent environmental conditions. Categorical variables included vegetation type (16 different types) (Abram et al., 2014) and habitat type (forest and oil palm), while continuous variables were represented by a set of light detection and ranging (LiDAR) images on elevation, slope, canopy height. Because of the high resolution of LiDAR images (1 m), elevation was used as proxy to the presence of water bodies and areas with different likelihood of flooding.

Unpublished data suggest that the diet of Asian water monitor population in the study site comprises a broad number of species among mammals, reptiles, amphibians, and invertebrates, with high proportion of rodents in oil palm, and no evidence of human‐made food. Hence, a subsample of 10 home ranges (*n*_Forest_ = 5; *n*_Oil palm_ = 5), divided into core and transient ranges was selected to perform the potential prey availability survey. Eight pitfall traps per site were used for invertebrates, reptiles, and amphibians, while 20 wire cage traps were deployed for small mammals. Surveys were carried out during nine trap nights per site, starting right after the GPS tracker was retrieved from the target individual, to avoid interference during the tracking period. Pitfall traps consisted of lines of two 20‐L plastic buckets (height 390 mm, top width 320 mm, bottom width 270 mm), with 12 m of 50 cm high plastic canvas drift fencing in total, and were checked twice daily at 08:00 and 15:00 h. Small mammal traps were also checked twice daily at 08:00 and 15:00 h.

### 2.5 Data analyses

Home range differences between plantation and forest were evaluated using a general linear model (GLM), while habitat preferences were analyzed with the MIAMaxent v.1.1.0 package for R (Vollering et al., 2019). The package is based on the maximum entropy (MaxEnt) algorithm for presence‐only data to evaluate the influence of multiple environmental variables on the distribution of any species and predicts the potential distribution of the species in a larger area (Halvorsen, 2013; Halvorsen et al., 2015; Phillips et al., 2006). The model was validated with the estimation of the area under the curve (AUC) where AUC < 0.5 was considered satisfactory (Phillips et al., 2006), and the suitable habitat was described as the area with high probability ratio of occurrence (PRO > 1). A percentage of it was calculated within each type of habitat (forest v. plantation), as well as per home range, for it to be included in further analysis.

For the potential prey availability, analysis of species richness was carried out at the lowest taxonomic level possible. Species abundance was compared between core and transient ranges, as well as between plantation and forest habitats. All identified taxa were categorized into three prey groups: Mammalia, Amphibia/Reptilia, and Invertebrates. Diversity was estimated using the Shannon Diversity Index (H′) in the “BiodiversityR” v.2.12‐1 (Kindt & Coe, 2005) and “vegan’ v.2.5‐6 (Oksanen et al., 2017) R packages. Simple linear models were used to assess differences among the different community index per habitat (forest v. oil palm) and range (core v. transient ranges) in R (Zuur et al., 2009).

Finally, we assessed the effect of different variables, including the percentage of suitable habitat within the polygon, as well as prey abundance and diversity (overall and per taxonomic group), in both the transient and core ranges. General Estimation Equations (GEE) models were run with the “geepack” package v 1.3‐2 for R (Halekoh et al., 2006; Hardin & Hilbe, 2002). One set of seven models were tested for each range using different combinations of the variables. All the variables were log10 transformed and scaled, the family error was set to Gaussian with an identity link function, the autocorrelation was defined as unstructured, and the variable “habitat” was used as group ID. Contrary to GLM, that need to estimate a within‐group variance component, GEE models estimate the average group response (i.e., habitat, study area, and study site) independently of the correlative structure between the groups (Yan & Fine, 2004; Zuur et al., 2009). However, as GEE models cannot use the known Akaike index criterion as a validation method, we used the adapted method QIC, as suggested by Pan (2001).

### 2.6 Ethics statement

Animal trapping, handling, and tagging protocols were designed and carried out by a certified veterinarian, in accordance with animal welfare guidelines from the National Centre for Replacement, Refinement and Reduction of Animals in Research. Protocols were reviewed and authorized by Sabah Wildlife Department and the Sabah Biodiversity Centre, as part of the procedures to authorize access to natural resources (permit number JKM/MBS.1000‐2/2JLD.3‐7). Felda Global Ventures Bhd. Malaysia and Ladang Kinabatangan Bhd. kindly granted the permits to perform our research in their plantation estates.

## 3 RESULTS

Out of the 20 tagged lizards, only 14 (*n*_Forest_ = 7; *n*_oil palm_ = 7) were included in the analyses. The remaining six individuals did not provide sufficient data to stabilize a home range size (less than 30 performing days and/or less than 150 GPS locations). Home range (LoCoH‐95) in forested areas ranged from 0.366 km^2^ to 1.292 km^2^ (0.879 ± 0.161), while in oil palm plantations, it varied from 0.066 km^2^ to 0.742 km^2^ (0.305 ± 0.095; *t* = −3.065; *p* = .009). Buffer areas (LoCoH‐75) measured 0.150 km^2^ to 0.520 km^2^ (0.319 ± 0.07) in forest, and 0.04 km^2^ to 0.360 km^2^ in oil palm plantations (0.119 ± 0.045; *t* = −2.398; *p* = .034). Core area (LoCoH‐50) estimations in forested areas ranged from 0.046 km^2^ to 0.203 km^2^ (0.134 ± 0.025), and from 0.001 km^2^ to 0.134 km^2^ in oil palm plantations (0.053 ± 0.019; *t* = −2.528; *p* = .026) (Table 1; Figure S1).

**TABLE 1:** Home range (LoCoH) estimations for Asian water monitor lizards in the Kinabatangan floodplain

ID	Habitat	LoCoH‐95	LoCoH‐75 (%)	LoCoH−50 (%)
T‐01	Forest	1.244	0.590 (47.4)	0.203 (16.3)
T‐02		0.366	0.224 (61.3)	0.163 (44.6)
T‐03		1.389	0.416 (30.0)	0.189 (13.6)
T‐04		0.673	0.150 (22.2)	0.053 (7.9)
T‐05		0.420	0.172 (41.0)	0.102 (24.2)
T‐06		1.292	0.520 (40.2)	0.180 (14.0)
T‐07		0.771	0.165 (21.5)	0.046 (5.90)
T‐08	Oil palm	0.066	0.004 (5.40)	0.001 (2.20)
T‐09		0.553	0.170 (30.8)	0.119 (21.6)
T‐10		0.133	0.036 (27.4)	0.020 (14.8)
T‐11		0.151	0.062 (41.3)	0.020 (13.1)
T‐12		0.183	0.128 (70.1)	0.044 (24.1)
T‐13		0.313	0.077 (24.7)	0.037 (11.7)
T‐14		0.742	0.360 (48.5)	0.134 (18.0)

Note

Areas are presented in km^2^, for three different levels of utilization. Percentages are proportional to LoCoH‐95.

**FIGURE 3:**
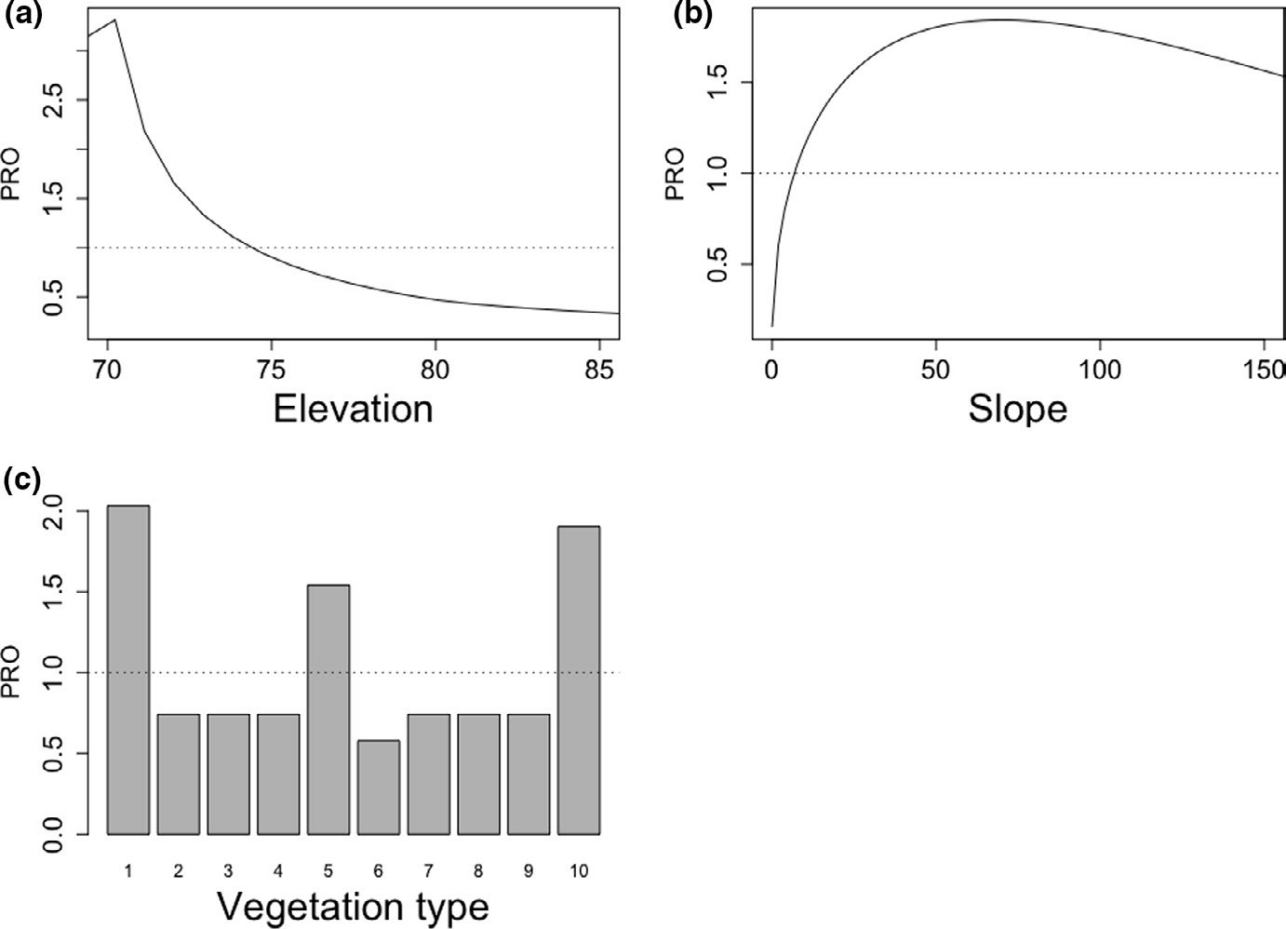
Probability ratio of occurrence (PRO) of Asian water monitor lizard according to (a) elevation, (b) slop, and (c) vegetation class. For vegetation class, variables correspond to: [1] severely degraded areas, [2] dry lowland forest, [3] limestone forest, [4] peat swamp forest, [5] seasonal freshwater swamp forest, [6] freshwater swamp forest, [7] swamp, [8] Cleared areas /young oil palm, [9] oil palm with good canopy, and [10] underproductive oil palm

**FIGURE 4:**
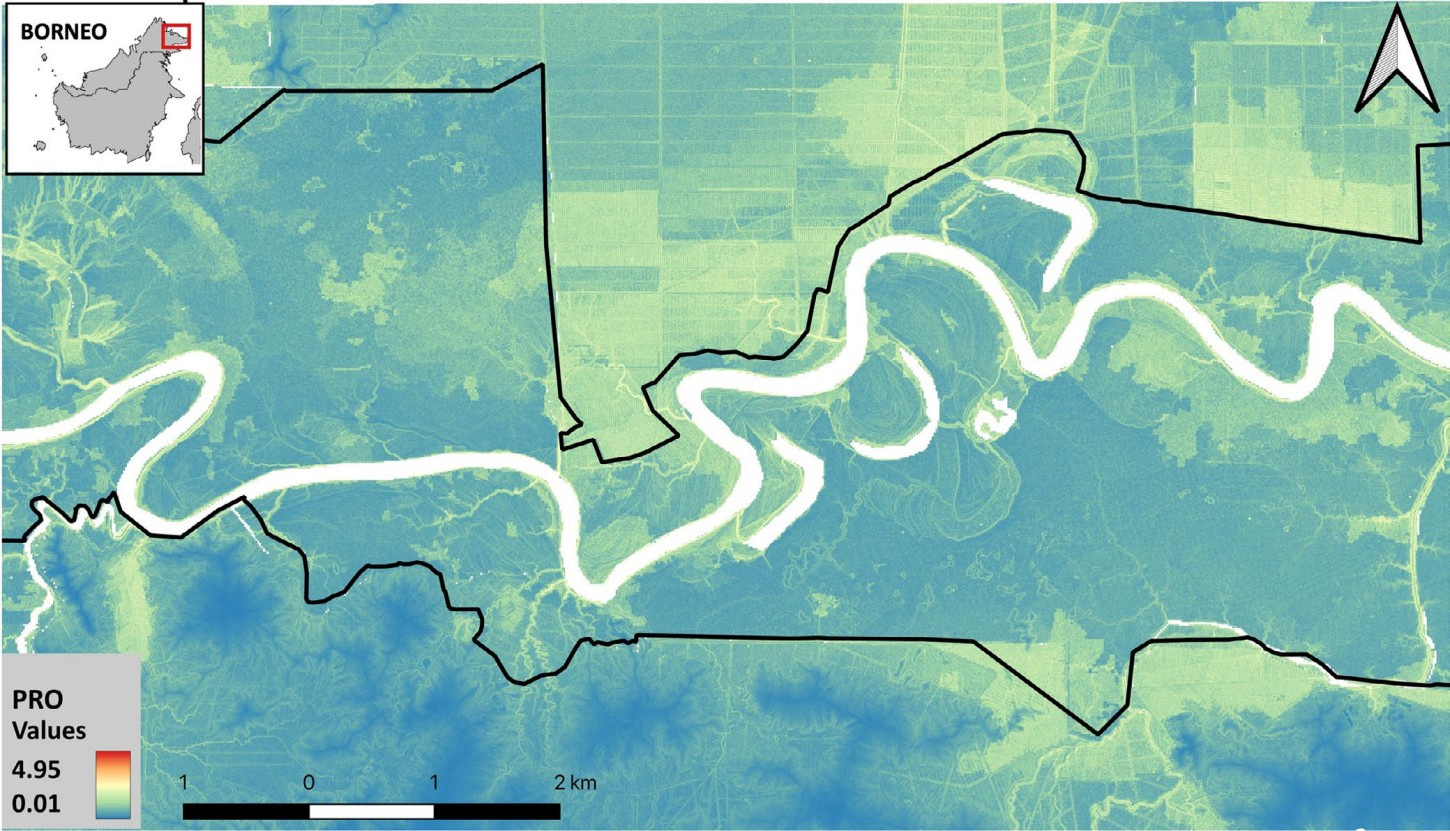
Representation of suitable areas for the Asian water monitor lizard population in the Kinabatangan floodplain. Gradient is determined by the probability ratio of occurrence (PRO), based on the presence of suitable environmental variables

Only elevation, slope, and vegetation type were identified as the most important variables influencing the distribution of Asian water monitor lizards within the study site (Figure 3; Table S1). The probability ratio of occurrence was higher (PRO > 1) in lower areas, with smooth slope, which are mostly related to temporal or permanent flooded sites, such as swamps, rivers, or lake shores (Figure 4). The analysis also revealed that vegetation type is an important factor for monitor lizard distribution, even more so than habitat type or forest structure (i.e., canopy height). Habitats that are severely degraded, or underproductive oil palm plantations, followed by seasonal freshwater swamp forest were the most determinant for monitor lizard distribution (PRO > 1.5) (Figure 3).

The model was appropriately validated (AUC = 0.771), and a predictive model of suitable habitat for the monitor lizard population was built (Figure 4). Forested areas hold a larger proportion of suitable area, largely distributed (31.71 out of 43.02 km^2^ [73.7%]), while oil palm plantation only hold 39.6% of suitable area for the species (16.49 out of 41.65 km^2^), restricted to clusters with water bodies and riparian vegetation with dense understory.

In the assessment of potential prey availability, we collected a total of 1,519 records representing 27 taxonomic families and 49 species after 2,520 trap‐nights (1,800 for the small mammals and 720 for pitfall traps). We found higher relative abundance (RA) of mammals in forested areas (RA_Forest_ = 0.02 ± 0.002; RA_Oilpalm_ = 0.008 ± 0.002; *F* = 3.533; *p* = .03), as well as higher diversity (H′_Forest_ = 1.674 ± 0.08; H′_Oilpalm_ = 0.849 ± 0.19; *F* = 5.22; *p* = .01). Although invertebrate abundance was not different between habitats, diversity was higher in forested areas (H′_Forest_ = 1.564 ± 0.079) than on oil palm plantations (H′_Plantation_ = 0.81 ± 0.133; *F* = 23.5; *p* < .001). Species diversity was significantly higher in forested areas in both core (H′ = 2.43 ± 0.073) and transient (H′ = 2.42 ± 0.086) ranges, compared with oil palm plantations (H′_Core_ = 1.53 ± 0.15; H′_Transient_ = 1.46 ± 0.14; *F* = 9.995; *p* = .0006). However, there were no differences for the overall abundance. The amphibian/reptile's group did not show differences for either relative abundance or diversity (Table 2).

**TABLE 2:** Potential prey availability per habitat (forest v. oil palm plantation), and type of range (Transient [LoCoH‐95] v. Core [LoCoH‐50]), presented as relative abundance and diversity index (H′) for mammals, reptiles/amphibians, and invertebrates

Habitat	Range	Relative Abundance (SE)			
		Species	Mammals	Reptiles/Amphibians	Invertebrates
Forest	Core	0.080 (0.006)	0.021 (0.003)	0.005 (0.001)	0.053 (0.008)
	Transient	0.067 (0.004)	0.019 (0.002)	0.004 (0.001)	0.039 (0.003)
Oil palm	Core	0.150 (0.078)	0.009 (0.003)	0.003 (0.001)	0.137 (0.075)
	Transient	0.113 (0.054)	0.007 (0.002)	0.004 (0.002)	0.102 (0.051)

Habitat	Range	Diversity H′ (SE)			
		Species	Mammals	Reptiles/Amphibians	Invertebrates
Forest	Core	2.428 (0.073)	1.711 (0.101)	0.456 (0.146)	1.551 (0.038)
	Transient	2.418 (0.086)	1.643 (0.071)	0.497 (0.157)	1.576 (0.113)
Oil palm	Core	1.529 (0.155)	0.985 (0.169)	0.227 (0.099)	0.692 (0.157)
	Transient	1.458 (0.145)	0.714 (0.220)	0.521 (0.169)	0.922 (0.110)

Mammals were represented by 24 species in forested areas and 11 in plantations. Six species identified in plantations belonged to the genus *Rattus* spp., from which the brown rat, *R*. *norvegicus* was only found in plantation habitat (Figure S2). The group of amphibians and reptiles was represented by eight species in forested areas, while we recorded only four species in plantations, where the most abundant were the frogs from the family Hylidae (*F* = 4.501; *p* = .030) (Figure S3). We found 15 species of invertebrates in forest, with high relative abundance of the family Myriapoda (*F* = 3.77; *p* < .001), while in plantations, there were only eight species, where ground beetles from the genus *Pheropsophus spp*. were the most abundant (*F* = 3.011; *p* = .014), especially in core ranges (Figure S4).

To evaluate the effect of different variables on the home range size, a total of seven GEE models were tested for both transient and core areas and ranked according to the QIC value (Table S2). For the transient areas (LoCoH‐95), the best model (QIC = 5.84) considered six different variables, including the percentage of suitable area within the home range, diversity index of species, and abundance and diversity of both invertebrates and mammals. Regarding to core areas, the best model (QIC = 6.13) only considered percentage of suitable habitat and abundance of species overall and separately (mammals and invertebrates).

We observed significant effects on the size of transient ranges by diversity index of the overall species (Wald = 16.70; *p* < .0001), invertebrates (Wald = 8.54; *p* = .003), and mammals (Wald = 41.01; *p* < .0001), as well as by the abundance of mammals only (Wald = 354.77; *p* < .0001). However, only mammal abundance (Wald = 7; *p* = .008) and the proportion of suitable habitat (Wald = 5.95; *p* = .015) showed significant effects on the size of core ranges (Table 3).

**TABLE 3:** Results of the GEE models on the influence of different variables on the size of the Asian water monitor lizards home range

LoCoH‐95				
Predictor	Estimate	SE	Wald	*p*
Intercept	0.862	0.020	1,928.440	<.001
Suitable habitat	−0.025	0.030	0.700	.401
Species H′	**2.035**	**0.498**	**16.700**	**<.001**
Invertebrate abundance	−0.152	0.054	7.750	.005
Invertebrates H′	−**0.839**	**0.287**	**8.540**	.**003**
Mammal abundance	**0.552**	**0.029**	**354.770**	**<.001**
Mammals H′	−**1.552**	**0.024**	**41.010**	**<.001**

LoCoH‐50				
Predictor	Estimate	SE	Wald	*p*
Intercept	**0.997**	**0.001**	**5253.210**	**<.001**
Suitable habitat	−**0.012**	**0.005**	**5.950**	.**015**
Species abundance	−0.037	0.040	0.850	.357
Invertebrate abundance	−0.008	0.034	0.050	.821
Mammal abundance	**0.026**	**0.010**	**7.000**	.**008**

Note

GEE results are for transient (LOCoH‐95) and core areas (LoCoH‐50). All the predictors were scaled. Significant *p* values are in bold.

## 4 DISCUSSION

The present study describes how the Asian water monitor lizard persists in a highly fragmented landscape in Northern Borneo by efficiently reducing their home range when inhabiting oil palm plantations, using areas of high prey abundance, and suitable environmental features. The robustness of the GPS data and the prey inventory within the home ranges provides valuable information about the monitor lizard population and their prey species′ ecology. Additionally, this study provides insight into the composition and distribution of the potential prey community available in oil palm plantations, which may contribute to the understanding and management of this human‐modified landscape.

A species′ home range size and shape are determined by the abundance of resources present in a given area (Gehring & Swihart, 2003; Saïd & Servanty, 2005). Contrary to specialist, generalist species can overcome barriers in human‐dominated landscapes, because of their plasticity in adaptation to such habitats (Gehring & Swihart, 2004; Swihart et al., 2003). As a generalist, it appears that the Asian water monitor lizard populations have benefitted from the expansion of industrial oil palm crops (Traeholt, 1997; Uyeda, 2009). However, a closer examination reveals that the resulting impacts of oil palm development on monitor lizard home ranges may influence the drastic shift in the population distribution, described by Guerrero‐Sanchez et al. (2021). Such impact is based not only on the altered prey community found in developed areas but also on the limited availability of suitable habitat, with potential consequences on both the population health and the prey community composition.

We observed that home ranges were larger when they include forest habitat within them, either totally or partially, while those set in only oil palm plantations were significantly smaller. Our Maxent analysis showed that the suitable habitat in plantations is reduced and restricted to areas close to water bodies (i.e., drains, swamps), with dense riparian understory and underproductive oil palm lots. These underproductive zones are described as areas with less than 25% of the average fruit production (Abram et al., 2014) and characterized by low human‐activity, as well as by frequent flooding events (pers. obs.). Our GEE models showed that the limited (and clustered) availability of suitable habitat limits the size and distribution of core home ranges in oil palm plantations, also associated with the abundance of small mammals.

We found that the monitor lizards in oil palm plantations possess only one small core area, apart from a single individual who was recorded to have two core areas, although within close range to one another. In contrast, monitor lizards inhabiting natural forests had a greater number of core ranges, which were also larger in size. Although the core ranges were attached to main water bodies, it is evident that the forest provides more suitable habitat for protection so lizards venture away for their water sources for a more even utilization of their home range.

While core ranges provide individuals with adequate protection and sufficient abundance of food, the size of the transient ranges rely basically on the protection offered, so an individual can move from one core range to another with low or null exposure to dangers (Auffenberg, 1981; Gehring & Swihart, 2003; Saïd & Servanty, 2005). The scattered distribution of suitable habitats within the forest allows monitor lizards to explore and establish different core areas within their home range, which may optimize the use of resources under the protection of the forest. Meanwhile, in plantations, they seem more comfortable staying in the same cluster, instead of venturing to other potentially suitable sites, as an attempt to avoid antagonist encounters with competitors, as well as to be exposed to unfavorable conditions, such as heat stress in open, sunny areas (Dawson, 1975; Huey, 1991; Wikramanayake & Dryden, 1993).

Prey composition is also demonstrably diverse in several varanid species, with rodents, birds and bird eggs, small reptiles, and amphibians, as well as a small percentage of invertebrates found in the majority of studied individuals (Jessop et al., 2012; Kulabtong & Mahaprom, 2015). Moreover, we found that monitor lizards in oil palm plantations feed on a nearly homogeneous diet (~80% rodent species), while those in forest have a broader range of prey in their menu (unpublished data). Same unpublished information suggests that birds might not play a relevant role as part of the diet in the study area, while fish presence was very low compared with other prey items. Hence, we did not consider those taxonomic groups for the potential prey assessment. However, we recommend the reader to take this into consideration before jumping into conclusions, as diet composition may differ in other regions.

Although our results did not show significant differences in the overall species abundance among habitats, mammals were significantly more abundant and diverse within the home ranges in forest than in oil palm plantations. However, the high relative abundance of rodents from the genus *Ratus* spp., especially the abundance of brown rats (*R*. *norvegicus)*, may compensate for the lower prey species biomass provided in oil palm plantations. Unfortunately, our study did not include the biomass assessment, and we suggest that further studies should include these data when assessing food availability.

In anthropogenic landscapes, generalist species can establish their home ranges in the boundaries between crops and forested areas in order to reduce the cost‐effect between food and protection (Gehring & Swihart, 2004; Saïd & Servanty, 2005). Our results from the GEE models suggest that the size of Asian water monitor's core range is larger where the abundance of mammals and amount of suitable habitat are high, while transient ranges are larger where mammals are less abundant. Auffenberg (1981) suggested that around 50% of the activities of Komodo dragons happen within the core range, which has specific features that makes it differ from less utilized areas. Although the size of core ranges relies on the abundance and distribution of resources that the Asian water monitor needs, the establishment of large transient ranges demand a large amount of energy, and it becomes necessary to find a balance between the amount of resources available and the size of the home range. Therefore, lower abundance of prey in the area will force the monitor lizard to increase its home range to ensure enough prey availability. However, in oil palm plantations, the amount of suitable habitat also plays a fundamental role on the size of core ranges, preventing the individuals of roaming beyond these areas, and exposing themselves to unnecessary risks.

The Asian water monitor lizard is one of the few species that has persisted despite the expansion of oil palm plantations in Borneo, because of its broad dietary requirements and high tolerance to human‐dominated landscapes (Twining et al., 2017). However, our findings suggest that this anthropogenic landscape is actually altering natural patterns of home range development in the Kinabatangan floodplain population, creating a sort of ecological trap that satisfies the needs of the individuals within smaller areas. Nonetheless, these conditions may not remain favorable for them in the long run. The reduced and clustered distribution of suitable habitat in oil palm plantations might not be a problem for large individuals, but it may have negative impact on the survival of juveniles and hatchlings, because of predation and competition, as well as microclimatic conditions (Guerrero‐Sanchez et al., 2021).

As oil palm plantations have become the dominant habitat type in the Bornean landscape, forest connectivity is imperative for the survival of many native species, including the Asian water monitor lizard. Such connectivity is important and can be achieved by the restoration of degraded forest, as well as by creating corridors or steppingstones within the plantation estates, especially in underproductive areas. The identification and assessment of these resourceful areas within the plantations could contribute to the design of a healthier landscape matrix and improve the chances of survival for many species with null or minimum impact on the productivity of the industrial crops.

To our knowledge, this study represents the first on the Asian water monitor lizard's spatial ecology using GPS technology with consideration of prey abundance as a variable. Hence there are several limitations that are worth considering in further studies. Firstly, the bias in terms of body size of the sampled individuals, which is greatly because of to the size of the GPS trackers. The constant improvement on the efficiency of GPS technology may improve the knowledge on how the species uses the landscape, by allowing the tagging of smaller individuals. Secondly, spatial imagery provided by drone technology may provide a better understanding of the use of the resources in real time. Third and lastly, biomass calculation of prey would provide more detailed information on how prey abundance influences the size and distribution of the home range. However, regardless the mentioned limitations, our findings highlight the impact of oil palm‐dominated landscapes on the dynamics of a generalist carnivore in Borneo, as well as the importance of generate a larger understanding on the dynamics of the animal community within oil palm habitats. It is advisable to encourage more studies in these anthropogenic habitats, in order to design more sustainable management strategies for the oil palm production.

## CONFLICT OF INTEREST

The authors declare no competing interests.

## AUTHOR CONTRIBUTIONS

**Sergio Guerrero‐Sanchez:** Conceptualization (lead); data curation (lead); formal analysis (lead); investigation (lead); methodology (lead); visualization (lead); writing – original draft (lead). **Katherine Majewski:** Formal analysis (supporting); methodology (equal); writing – original draft (supporting); writing – review and editing (equal). **Pablo Orozco‐terWengel:** Conceptualization (supporting); methodology (supporting); supervision (equal); writing – review and editing (equal). **Silvester Saimin:** Resources (supporting); writing – review and editing (supporting). **Benoit Goossens:** Conceptualization (supporting); formal analysis (supporting); funding acquisition (lead); investigation (supporting); methodology (supporting); project administration (equal); resources (lead); supervision (lead); writing – review and editing (equal).

## Supporting information

Appendix S1

## Data Availability

The data that support the findings of this study are available on Dryad at https://doi.org/10.5061/dryad.f4qrfj6xb.
