# Supplementary material for: The effect of oil palm‐dominated landscapes on the home range and distribution of a generalist species, the Asian water monitor
Source: Ecol Evol. 2022 Jan 26;12(1):e8531. doi: 10.1002/ece3.8531 (PMC8794710; doi:10.1002/ece3.8531)
Supplement: Supplementary file 1 — Appendix S1 [file ECE3-12-e8531-s001.pdf]

The effect of oil palm dominated landscapes on the home range and distribution of a generalist species, the Asian water monitor.

Supplementary material

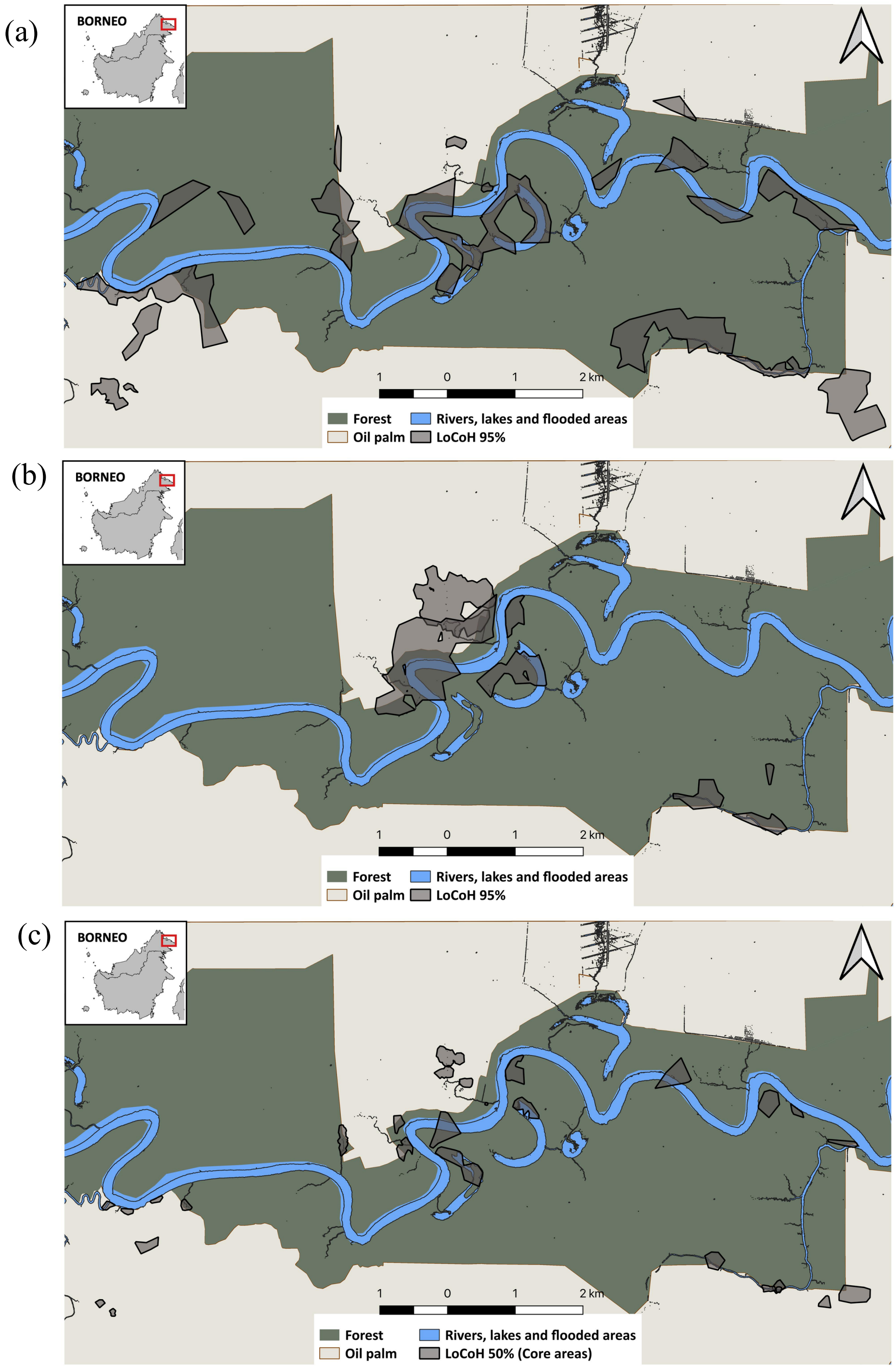

Fig. S1. Estimated home range for 14 Asian water monitor lizards in the Kinabatangan floodplain. Home ranges correspond to local convex hull (LoCoH) estimations for 95% (maps [a] and [b]), and 50% (map [c]).

Table S1. Influence of the environmental variables on the Asian water monitor lizard distribution within the study area, standalone and in combination with each other, either by addition or interaction.

Environmental variables	m	Dsq.	Xi^2	df	p
Vegetation type	4	0.018	2102.483	4	0
Elevation	3	0.011	1338.135	3	7.82E-290
Slope	2	0.01	1215.574	2	1.10E-264
Habitat	1	0.001	169.967	1	7.52E-39
Canopy height	1	0.001	105.816	1	8.09E-25
Vegetation type + Slope	6	0.025	813.766	2	1.96E-177
Vegetation type + Elevation	7	0.024	749.851	3	3.25E-162
Vegetation type + Habitat	5	0.018	6.613	1	0.0101
Vegetation type + Canopy height	5	0.018	0.8144	1	0.367
Vegetation type + Slope + Elevation	9	0.033	968.167	3	1.45E-209
Vegetation type + Slope + Elevation + Vegetation type: Slope	17	0.035	240.032	8	2.23E-47
Vegetation type + Slope + Elevation + Vegetation type: Elevation	21	0.034	137.738	12	1.71E-23
Vegetation type + Slope + Elevation + Slope: Elevation	15	0.034	82.341	6	1.17E-15
Vegetation type + Slope + Elevation + Vegetation type: Slope + Vegetation type: Elevation	29	0.036	105.341	12	4.97E-17
Vegetation type + Slope + Elevation + Vegetation type: Slope + Slope: Elevation	23	0.035	38.926	6	7.40E-07
Vegetation type + Slope + Elevation + Vegetation type: Slope + Vegetation type: Elevation + Slope: Elevation	35	0.036	36.003	6	2.75E-06

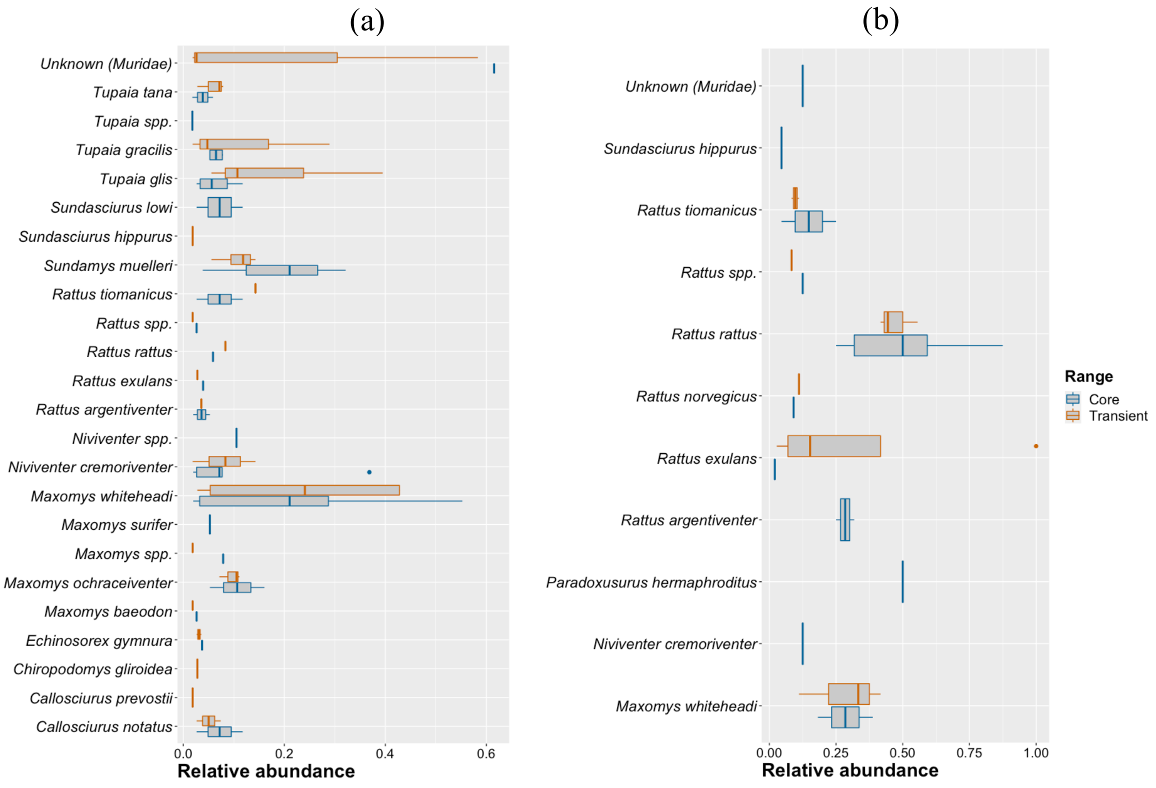

Fig. S2. Relative abundance of species (mammals) identified in (a) forest and (b) oil palm areas and compared between core and transient ranges determined by the number of sampling sites established in the study.

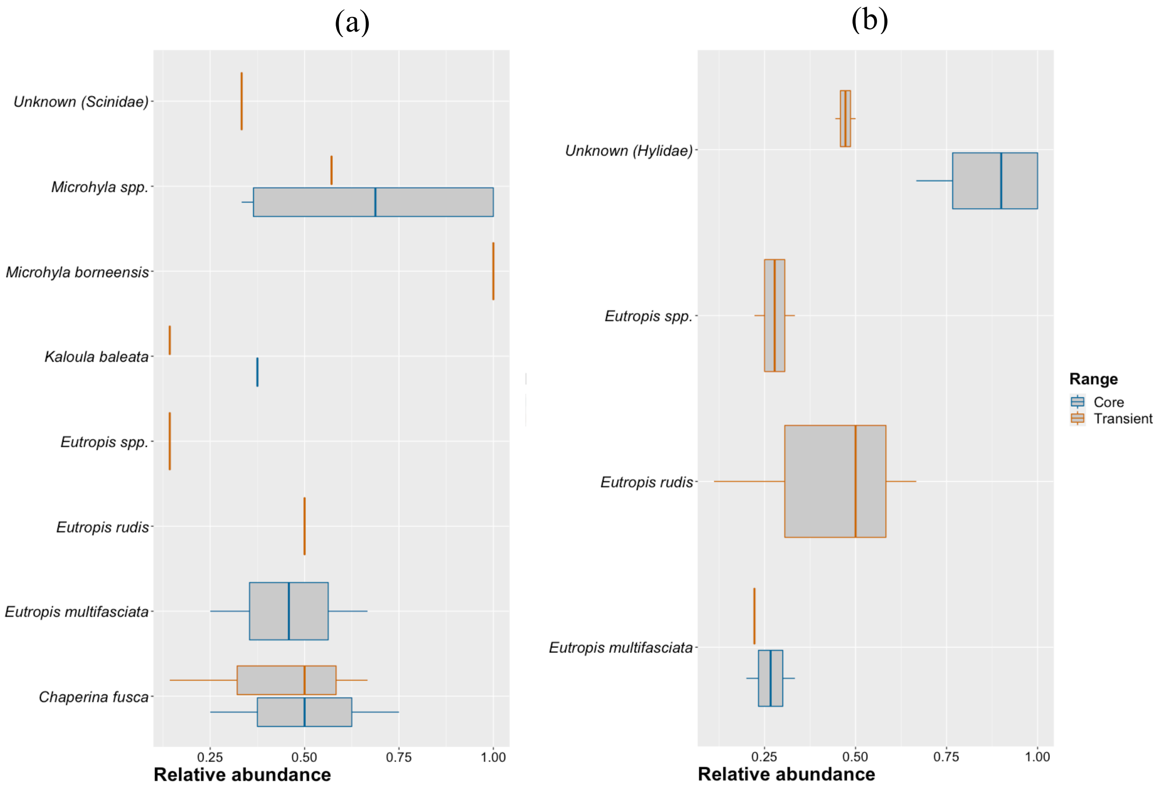

Fig. S3. Relative abundance of species (reptile and amphibian) identified in (a) forest and (b) oil palm areas and compared between core and transient ranges determined by the number of sampling sites established in the study.

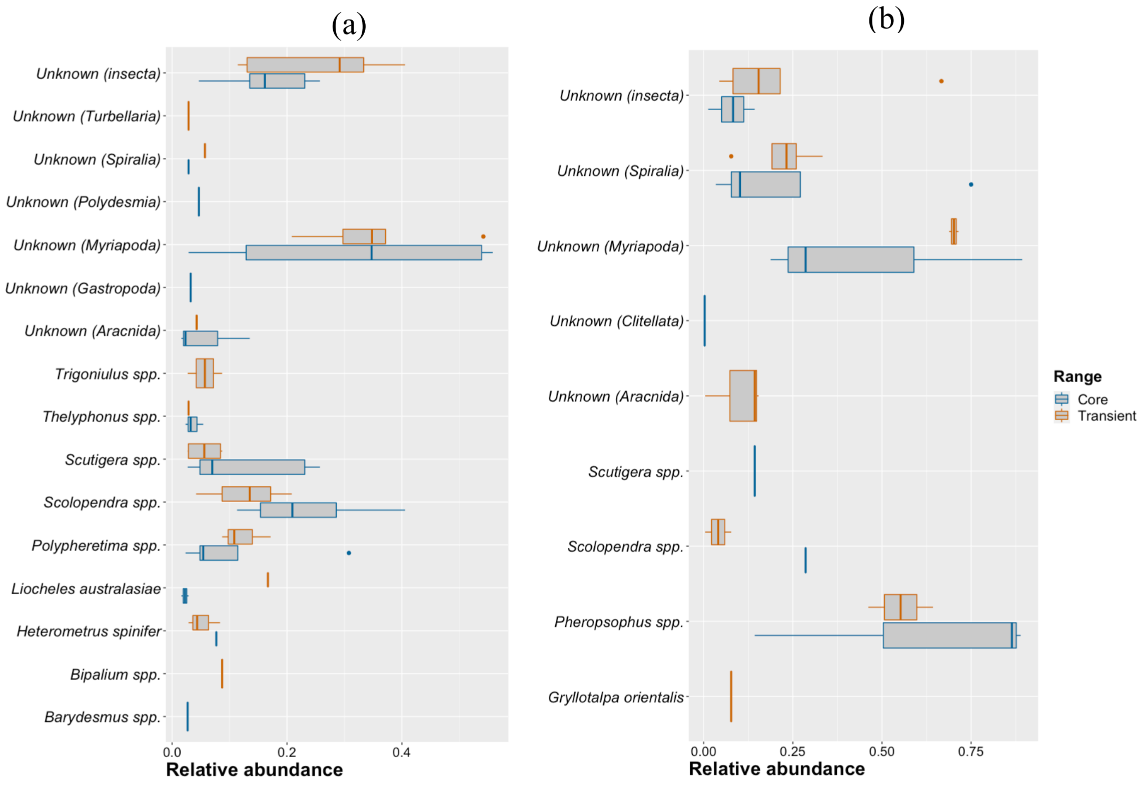

Fig. S4. Relative abundance of species (invertebrates) identified in (a) forest and (b) oil palm areas and compared between core and transient ranges determined by the number of sampling sites established in the study.

Table S2. Tested GEE models to assess the influence of different variables on the size of transient (LoCoH-95) and core (LoCoH-50) areas of the home range of Asian water monitor lizards. Model were ranked according with the QIC value.

							LoCoH-95						
**Model**	**(Intercept)**	**Suit. Hab.**	**sp. Ab.**	**sp. H'**	**Inv. Ab.**	**Inv. H'**	**Mam. Ab.**	**Mam. H'**	**df**	**qLik**	**QIC**	**delta**	**weight**
**M- 4**	0.862	-0.025	NA	2.035	-0.152	-0.839	0.552	-1.552	NA	-5.000	5.848	0.000	0.375
**M- 2**	0.682	0.050	1.888	NA	-2.151	NA	0.067	NA	NA	-5.000	6.171	0.323	0.319
**M- 6**	0.834	0.459	NA	-0.283	-0.423	NA	0.218	NA	NA	-5.000	6.960	1.112	0.215
**M- 1**	0.651	-0.028	-2.847	2.741	2.572	-1.296	0.561	-1.478	NA	-5.000	8.687	2.839	0.091
**M- 5**	0.962	0.206	0.377	NA	-0.375	0.689	0.124	-0.863	NA	-5.000	46.307	40.458	0.000
**M- 3**	0.101	1.720	NA	-2.037	NA	2.693	NA	-0.801	NA	-5.000	57.957	52.108	0.000
**M- 7**	0.508	1.083	-2.399	NA	NA	-2.625	NA	2.833	NA	-5.000	416.308	410.460	0.000

							LoCoH-50						
**Model**	**(Intercept)**	**Suit. Hab.**	**sp. Ab.**	**sp. H'**	**Inv. Ab.**	**Inv. H'**	**Mam. Ab.**	**Mam. H'**	**df**	**qLik**	**QIC**	**delta**	**weight**
**M- B**	0.100	-0.012	-0.037	NA	-0.008	NA	0.026	NA	NA	-5.000	6.130	0.000	0.293
**M- D**	0.101	0.018	NA	-0.026	-0.027	0.062	-0.002	0.006	NA	-5.000	6.322	0.192	0.266
**M- G**	0.094	0.054	NA	0.071	0.038	NA	-0.058	NA	NA	-5.000	7.196	1.066	0.172
**M- C**	0.100	0.012	NA	0.018	NA	0.037	NA	0.005	NA	-5.000	7.317	1.187	0.162
**M- A**	0.100	0.034	0.182	-0.034	-0.145	0.081	-0.088	0.017	NA	-5.000	8.473	2.343	0.091
**M- E**	0.112	0.040	0.228	NA	-0.158	0.070	-0.116	-0.004	NA	-5.000	11.723	5.593	0.018
**M- F**	0.030	0.151	-0.034	NA	NA	0.065	NA	-0.045	NA	-5.000	832.988	826.859	0.000

Suit. Hab.= percentage of suitable habitat within the home range; sp. Ab.= Species abundance; sp. H'= species diversity; Inv. Ab.= Abundance of Invertebrates; Inv. H'= Invertebrates diversity; Mam. Ab.= Abundance of mammals; Mam. H' = mammals diversity.

Description of the performance of the GPS / VHF tags on Asian water monitor lizards

During this study, we tagged a total of 20 individuals, However, six of them were discarded due to the little amount of information provided by the tags, especially due to problems with the attachment system. But they provided valid information that help us to improve the devices for the rest of the animals.

Attachment belts for the first devices designed for this study were made of thick leather belts, with very little flexibility. The resulting attachment was therefore a bit loose, as we were cautious of causing skin abrasions, or affecting the lizards' movements and survival. This method however caused the tags to slip from the lizard's hips easily when they walked under tangled vines, logs and small burrows (spaces from where lost tags were later retrieved). A second and final generation of tag design was created with a more flexible (and smoother) rubber belt with a thinner leather core. It was also necessary that the device was smoothed at the front border, to allow the lizard to slide under vegetation such as vines without struggling. The maximum period of work logged for an individual was recorded over around 300 days, while the average performance of each tag was 154 days. The number of GPS locations recorded per lizard was relative to the number of days each lizard maintained the tracking device in position on its body, and the number of GPS locations expected to be logged per day, (11 GPS locations / day). The estimated success rate ranged between 75.16% and 19.7% (49.79% ± 4.66). The success rate for GPS fixing points in forest areas (with denser canopy cover) was calculated in 46% ± 4.61, while in plantations, it was estimated in 53% ± 4.73 with no significant differences ($F = 0.64$; $p = 0.43$). The following pictures show the two different type of belts and the attaching system onto the lizards (Fig S5).

Although the modifications we made improved the devices' functionality for the study of movement ecology of varanids, the reliance on two "AA" batteries to ensure long-term information make the tags relatively voluminous, limiting telemetry studies only to large individuals. Furthermore, although tags used in this study did not have a drop-off mechanism, lizards could be re-captured in order to retrieve the device, and no signs of injuries on the individuals were observed. This current model can last for enough time to get accurate data to answer some crucial questions on monitor lizards' spatial ecology. However, improving the devices in terms of size and durability could help to increase the knowledge of the ecology of this and other varanid species by including smaller individuals.

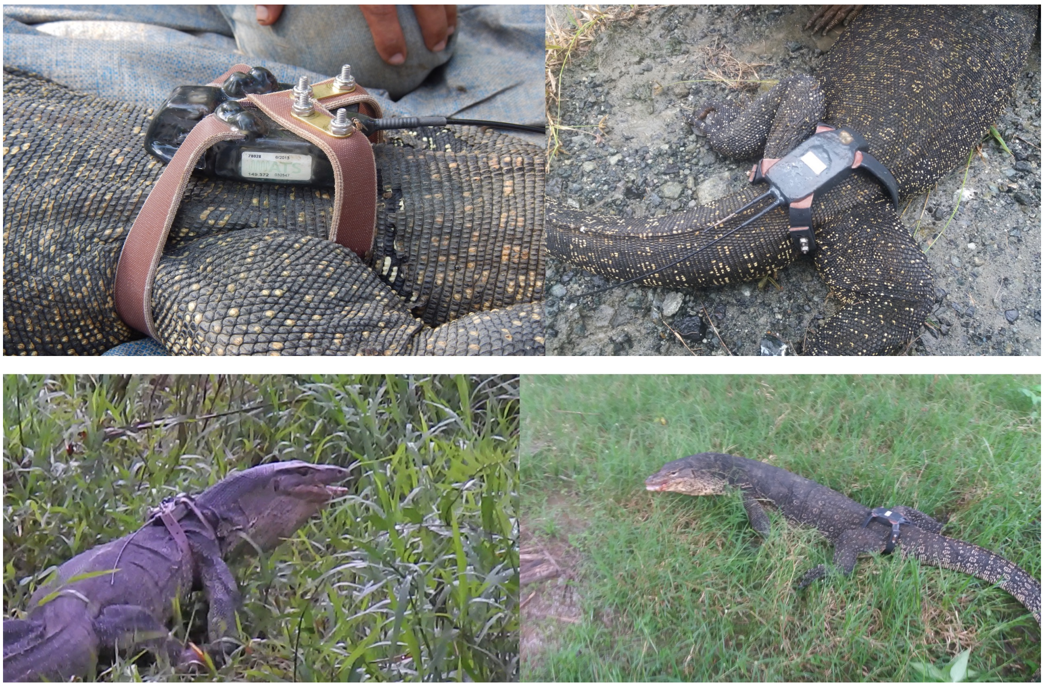

Fig. S5. VHF/GPS tags used on the Asian water monitor lizards during the study. The pictures above show the first (left) and second (right) generations of tags with the belts wrapped in rubber tube. The front of the device was smoothed for the second generation, to avoid entanglements. The pictures below show two different ways of attachment, being the hip (right) much better option than the shoulders region (left). Picture credits: Guerrero-Sanchez.
